# Structure-Guided Design of Planarized Catechin Derivatives: Enhancing Antioxidant and Multifaceted Biological Activities for Therapeutic Applications

**DOI:** 10.3390/antiox15080964

**Published:** 2026-08-01

**Authors:** Kiyoshi Fukuhara, Ikuo Nakanishi, Hiromu Ito, Wakana Shimizu, Yoshimi Shoji, Kei Ohkubo, Masao Morita, Sakurako Okada, Akiko Ohno

**Affiliations:** 1 Division of Organic and Medicinal Chemistry, Showa Medical University School of Pharmacy, Shinagawa-ku, Tokyo 142-8555, Japan; shimizu.wkn@cmed.showa-u.ac.jp (W.S.); masao.morita@showa-u.ac.jp (M.M.); ikeda-s@cmed.showa-u.ac.jp (S.O.); 2 Quantum RedOx Chemistry Team, Quantum Life Spin Group, Institute for Quantum Life Science (iQLS), National Institutes for Quantum Science and Technology (QST), Chiba-shi 263-8555, Japan; nakanishi.ikuo@qst.go.jp (I.N.); ito.hiromu@qst.go.jp (H.I.); shoji.yoshimi@qst.go.jp (Y.S.); ohkubo@irdd.osaka-u.ac.jp (K.O.); 3 Institute for Open and Transdisciplinary Research Initiatives (OTRI), Osaka University, Suita, Osaka 565-0871, Japan; 4 Division of Genome Safety Science, National Institute of Health Sciences, 3-25-26 Tonomachi, Kawasaki-ku, Kawasaki City 210-9501, Japan; ako-ohno@nihs.go.jp

**Keywords:** catechin, planarization, oxidative stress, glucosidase: antiviral activity, antitumor activity, amyloid-β, procyanidin, silybin

## Abstract

Oxidative stress is a major driver of chronic disease, making natural polyphenols attractive scaffolds for modulating inflammation, proteostasis, and cell fate. However, green-tea catechins possess a twisted, conformationally flexible flavan-3-ol framework that limits π-conjugation, phenoxyl-radical stabilization, and productive interactions with biological targets. This review presents a structure-based framework in which conformational planarization serves as a strategy for functional amplification. Preorganization of the A/C–B inter-ring bond into a nearly coplanar arrangement reduces the conformational entropy penalty (−*T*Δ*S*) upon binding or reaction while extending conjugation, strengthening π–π interactions, and facilitating redox reactions. This review highlights planarized catechin (PCat) architectures, including PCat–DTPA (a lesion-activated metal-responsive antioxidant), PCat–TrOH (a self-regenerating antioxidant network), procyanidin B3–PCat hybrids, and a planar silybin analog, illustrating how planarization and multivalent recognition enhance ROS regulation, inhibit amyloid-β aggregation and neurotoxicity, and suppress cancer cell phenotypes. Collectively, these studies establish PCat as a modular platform for the mechanism-informed design of disease-tailored phenolic antioxidants.

## 1. Introduction

Oxidative stress is currently known as a central etiological factor in the initiation and progression of major chronic diseases, including cancer [1], cardiovascular [2], cerebrovascular disorders [3], and diabetes [4], and in neurodegenerative conditions such as Alzheimer’s disease (AD) [5]. Excess formation of reactive oxygen species (ROS) and free radicals damages key biomacromolecules, such as lipids, proteins, and DNA, thereby triggering membrane peroxidation, proteostatic failure, mutagenesis, inflammatory signaling, and apoptosis [6,7,8,9]. Antioxidants function as endogenous and exogenous defense agents by terminating lipid peroxidation chain reactions and scavenging or reducing radical species [10,11]; accordingly, their relevance spans disease prevention and therapeutic intervention [12,13,14,15].

Most dietary or supplemental antioxidants are plant-derived, with polyphenols, carotenoids, and tocopherols being the prototypical classes. In addition to direct redox chemistry, these compounds modulate cell signaling pathways governing inflammation, cell death, and transcriptional control, thereby contributing to the maintenance of physiological homeostasis [16,17,18,19]. Polyphenols are particularly versatile; their diverse aromatic scaffolds and phenolic hydroxyl groups confer radical-quenching capacity and anti-inflammatory, antiviral, anti-allergic, antitumor, and neuroprotective activities [20,21,22,23,24,25]. In AD, multiple polyphenols attenuate amyloid-β (Aβ) aggregation and mitigate Aβ-evoked neurotoxicity [26,27], highlighting the value of agents that couple antioxidant function with protein-aggregation modulation. In oncology, a dysregulated redox balance supports proliferation and motility; ROS control by antioxidants can impose cell cycle arrest or promote apoptosis, thus contributing to tumor suppression [21,28].

Within this landscape, catechins, the major polyphenols in green tea, have been widely studied for their antioxidant and anti-inflammatory effects and for their neuroprotective and antiproliferative activity [29,30,31,32]. Catechins are flavan-3-ol derivatives whose phenolic hydroxyl groups participate in radical capture and, in some cases, metal chelation [33,34]. However, native catechins adopt twisted, non-planar conformations that limit inter-ring π-resonance [35,36]. This conformational flexibility imposes intrinsic ceilings on the electron-donating ability and stabilization of phenoxyl radical intermediates, thereby constraining electron transfer (ET) and hydrogen atom transfer (HAT) pathways that underlie phenolic antioxidant action [37,38,39,40]. These trends suggest that structural optimization to enforce planarity and tune the electronic structure is a practical and feasible approach for functional enhancement. Over the past two decades, extensive efforts have been devoted to the structural optimization of catechins in order to improve their biological activity, physicochemical properties, and pharmacokinetic behavior while preserving the flavan-3-ol framework [41,42]. Representative approaches include substitution at the 3-hydroxyl group to enhance lipophilicity and membrane permeability; functionalization at the C4 position to optimize interactions with biological targets [43,44]; and modification at the C8 position to improve antioxidant, anticancer, antimicrobial, antiviral, and anti-amyloid activities [45,46]. These strategies primarily rely on the introduction of functional groups that modulate physicochemical properties, electronic characteristics, or molecular recognition. In contrast, direct control of the three-dimensional conformation of the catechin scaffold itself has remained relatively unexplored. The concept of conformational locking through molecular planarization therefore represents a distinct structural optimization strategy that simultaneously modulates electronic structure, thermodynamic properties, and biological function.

Structure-guided solutions have emerged through the chemical rigidification and planarization of catechin-type frameworks. Conformational locking enhances hyperconjugative interactions and local electronic delocalization around the catechol redox center while reducing conformational entropy loss upon binding or reaction. In addition, the electron-donating alkyl bridge introduced during the locking process further increases the electron density of the catechol moiety, facilitating ET and HAT. Such rigidified molecules frequently display improved kinetic potency (faster radical trapping) and increased capacity (greater total radical equivalents), while enhancing the directionality and strength of noncovalent interactions (π–π stacking, hydrogen bonding, electrostatics) at biological interfaces.

In addition to the pure redox performance, this planarization strategy is modular and deployable across ROS-linked pathologies. In the context of AD, enforcing planarity within catechin derivatives or related polyphenol scaffolds can reinforce multivalent interactions with Aβ surfaces and divert self-assembly away from β-sheet-rich, neurotoxic aggregates, thereby alleviating downstream cellular stress [47,48,49]. In oncology, rigid catechin analogs can alter enzyme inhibition profiles and cellular pathways, thereby contributing to growth inhibition and reducing migratory capacity. This approach is compatible with hybrid designs; incorporating, for example, metal-chelating motifs enables disease-site-selective ROS control by sequestering catalytic iron and attenuating Fenton/Haber–Weiss chemistry, whereas conjugation to redox-active cofactors establishes intramolecular self-regenerating antioxidant networks.

This review focuses on molecular planarization as one of several structural optimization strategies that have been developed for catechin derivatives. Rather than providing a comprehensive survey of all catechin modifications, it specifically highlights how conformational locking of the flavan-3-ol scaffold enhances antioxidant activity and broadens biological functions through favorable electronic and thermodynamic effects. It first outlines the physicochemical principles governing phenolic antioxidant activity and the limitations imposed by nonplanar, flexible catechin conformations. It then summarizes advances in planarized catechins (PCat), in which conformational locking and π-conjugation expansion yield amplified radical-scavenging behavior. Finally, this review discusses how the electronic consequences of planarity, enhanced resonance stabilization, and strengthened directional noncovalent contacts translate into multifaceted clinical outcomes, including ROS control, inhibition of Aβ aggregation and neurotoxicity, and suppression of cancer-cell phenotypes. Taken together, these developments motivate a structure–function framework in which deliberate rigidification and modular derivatization of phenolic antioxidants provide a generalizable medicinal chemistry strategy for disease prevention and therapy.

## 2. Structural Chemistry of Catechins and Mechanistic Bases for Enhancing Antioxidant Activity

**Figure 1:**
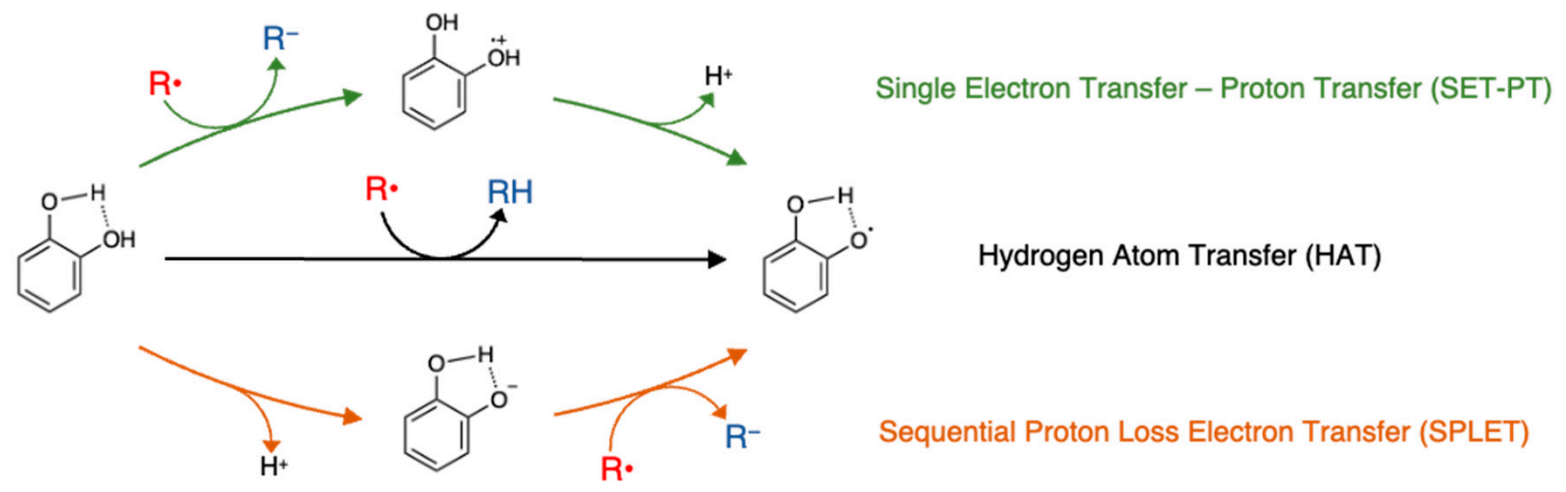
Radical scavenging mechanism of the catechol moiety. R•: reactive oxygen species or free radical.

Catechins are prototypical dietary polyphenols abundant in green tea and many fruits [29,50,51]. Chemically, they are flavan-3-ols bearing a tricyclic A/B/C framework, in which the B-ring 3′,4′-dihydroxy (catechol) unit is the principal determinant of the redox behavior [35,52,53]. Phenolic hydroxyls suppress ROS and organic radicals by donating a hydrogen atom or an electron. Because the two OH groups of a catechol are ortho, they engage in internal H-bonding and resonance assistance [40,54,55]. Moreover, their acidity (pKa ≈ 8–9) permits the formation of the corresponding phenolate (ArO^−^) under appropriate conditions [56]. As a result, catechin antioxidants operate through environment-dependent pathways (Figure 1) [57,58,59]. In nonpolar or neutral media, HAT from the phenolic O–H typically dominates, and the resulting ArO^•^ is markedly stabilized. In polar/protic media, single-electron transfer followed by proton transfer (SET–PT) is favored, wherein initial electron transfer from ArOH affords ArOH^•+^ that subsequently deprotonates. Under basic conditions or in the presence of coordinating metals, prior deprotonation to ArO^−^ enables sequential proton loss electron transfer (SPLET), often with reduced barriers for the ensuing ET step.

Across these regimes, the antioxidant efficacy is dictated by a small set of thermodynamic and electronic parameters. The O–H bond dissociation enthalpy (O–H BDE) governs the ease of H-donation in HAT; the oxidation potential (Eox) controls the facility of electron transfer in SET–PT and SPLET; and the extent of resonance stabilization of oxidized intermediates (ArO^•^, ArOH^•+^) determines their thermodynamic favorability and lifetime. Hence, the rational enhancement of catechin activity seeks to simultaneously lower BDE, decrease Eox, and broaden π-delocalization. Structure-based tactics that achieve these aims include installing electron-donating substituents (e.g., –R, –OH, –OMe, and –NR_2_) at para or ortho positions of the catechol ring to increase electron density and enforcing molecular planarity by conformational rigidification, which expands the conjugated network and reduces conformational entropy loss upon reaction or binding. Collectively, such modifications facilitate H-atom delivery and accelerate the initial ET steps, whereas enhanced delocalization stabilizes the phenoxyl and radical-cation species and channels the system toward thermodynamically benign oxidized states. Practically, the optimization of catechins depends on the coordinated tuning of bond energetics (BDE) and electronic delocalization (Eox), yielding simultaneous improvements in radical-quenching kinetics and capacity under physiologically relevant conditions.

It should be emphasized that the electronic effect of planarization is not attributed to uninterrupted π-conjugation across the flavan skeleton, because the B ring remains connected to the A/C framework through a saturated carbon atom. Rather, it is proposed that conformational locking enhances hyperconjugative interactions and local electronic delocalization around the catechol redox center by improving orbital alignment between the B-ring π orbitals and the adjacent σ-bond framework. In addition, the electron-donating alkyl bridge introduced during the conformational-locking process may further increase the electron density of the catechol moiety, thereby cooperatively lowering the oxidation potential and facilitating radical scavenging. This mechanistic interpretation is consistent with the experimentally observed decreases in oxidation potential and increases in radical-scavenging rate constants, although further computational and spectroscopic investigations will be required to quantify the relative contributions of hyperconjugation and the electron-donating substituent effect.

## 3. Planarization of Catechins by Conformational Locking

**Figure 2:**
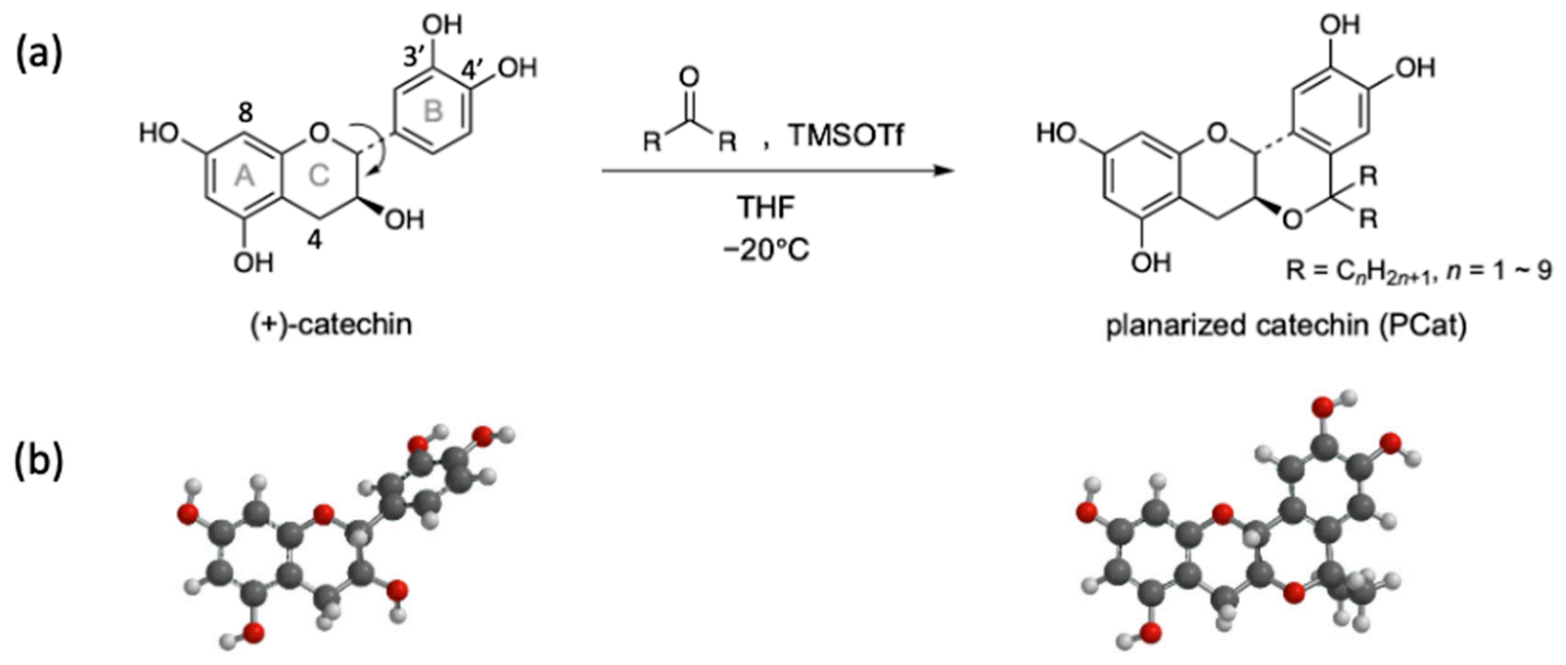
Conformational planarization of (+)-catechin. (**a**) Planarization via oxa–Pictet–Spengler (OPS) cyclization. R represents a linear C1–C9 alkyl group. (**b**) DFT-calculated lowest-energy structures of (+)-catechin and planar catechin (R = CH_3_).

Based on the thermodynamic and electronic considerations outlined above, PCat was engineered by conformationally locking (+)-catechin via ketone-mediated oxa–Pictet–Spengler (OPS) cyclization (Figure 2a) [60,61]. The natural catechin is dissolved in THF, treated with acetone and trimethylsilyl trifluoromethanesulfonate as a Lewis acid, and stirred at −20 °C for several hours, which installs an isopropyl tether that bridges the A–C and B rings with near-quantitative conversion [62]. The resulting scaffold exhibits high planarity and carries an isopropyl-derived electron-donating substituent para to the B-ring catechol, thereby increasing orbital overlap between the catechol π-system and neighboring σ-bonds within the rigidified scaffold. Although uninterrupted π-conjugation is not established across the entire flavan framework, the enforced geometry is expected to strengthen hyperconjugative interactions and facilitate local electronic delocalization around the catechol redox center (Figure 2b). This structural transformation has several mutually reinforcing effects. First, the O–H BDEs are reduced, facilitating HAT. Second, the oxidation potentials (E_ox_) decrease, accelerating the initial electron transfer step in SET–PT. Third, conformational rigidification promotes extensive delocalization of spin and charge over the planarized π-system, which stabilizes phenoxyl and radical-cation intermediates and suppresses back reactions. Consistent with these predictions, the radical-scavenging activity of the isopropyl-bridged PCat was first quantified using a model radical in acetonitrile, where its apparent radical-trapping reactivity was approximately fivefold greater than that of (+)-catechin. In a subsequent aqueous assay using β-cyclodextrin-solubilized DPPH^•^ in 0.1 M phosphate buffer at pH 7.4 and 298 K, the second-order rate constants were 9.6 × 10^3^ M^−1^ s^−1^ for PCat and 9.9 × 10^2^ M^−1^ s^−1^ for (+)-catechin, corresponding to an approximately 9.7-fold difference [63]. These results demonstrate that the OPS-derived analog reacts more rapidly than (+)-catechin in both non-aqueous and buffered aqueous model systems.

Because ^•^OH is the most reactive ROS and a primary driver of oxidative biomolecular damage and aging, the antioxidant performance was benchmarked in a Fenton system that cleaves supercoiled pBR322 DNA, an established model of oxidative injury [61]. (+)-Catechin attenuated DNA scission at high concentrations but paradoxically increased strand breaks at low concentrations, consistent with the pro-oxidant activation of Fenton chemistry. In contrast, PCat showed no pro-oxidant window and suppressed DNA cleavage across a broad concentration range, indicating a superior safety margin alongside higher efficacy [64].

The OPS platform is modular: replacing acetone (R = CH_3_) with other ketones enables the installation of diverse substituents via a bridging unit. Using this modularity, PCat analogs were designed with linear alkyl side chains (R = C*_n_*H_2*n*+1_, *n* = 2–9) to increase lipophilicity and bias reactivity toward lipid-phase peroxyl radicals, which are implicated in cardiovascular pathogenesis [62]. In the kinetic assays of ROO^•^ scavenging, the activity increased with chain length, peaked at *n* = 3–4, and then decreased for longer chains. An analogous inverted U was observed in the Fenton/pBR322 model: maximal suppression of DNA scission at *n* = 4–5, with diminished protection beyond that range. These trends underscore that planarity is necessary but not sufficient; fine control of peripheral substituents governs the phase behavior, encounter frequency with target radicals, and the microenvironment of HAT/ET events.

Collectively, OPS-based planarization preorganizes the catechin scaffold and optimizes its electronic structure, yielding a class of antioxidants with greater kinetic potency, enhanced radical-equivalent capacity, and reduced pro-oxidant activity. The synthetic handle provided by the ketone partner further enables ADME-oriented tailoring and targeting of specific radical milieus (e.g., lipid peroxidation), establishing PCat as a chemically tractable scaffold for next-generation antioxidant designs.

## 4. Thermodynamic Rationale for Planar Conformational Locking of Catechins

Rational drug design hinges on a quantitative understanding of binding thermodynamics at receptor and enzyme active sites, with the free energy of binding, Δ*G* = Δ*H* − *T*Δ*S*, governed by a delicate interplay between enthalpic (Δ*H*) and entropic (Δ*S*) terms. Hydrogen bonding, electrostatics, and π–π stacking contribute favorable enthalpy, whereas conformational restriction of the ligand and target, along with solvent reorganization, modulates the entropic balance. Therefore, achieving high affinity and selectivity requires not only strengthening directional interactions but also managing conformational and solvation entropy, an approach that has become central to modern medicinal chemistry in view of pervasive enthalpy–entropy compensation.

**Figure 3:**
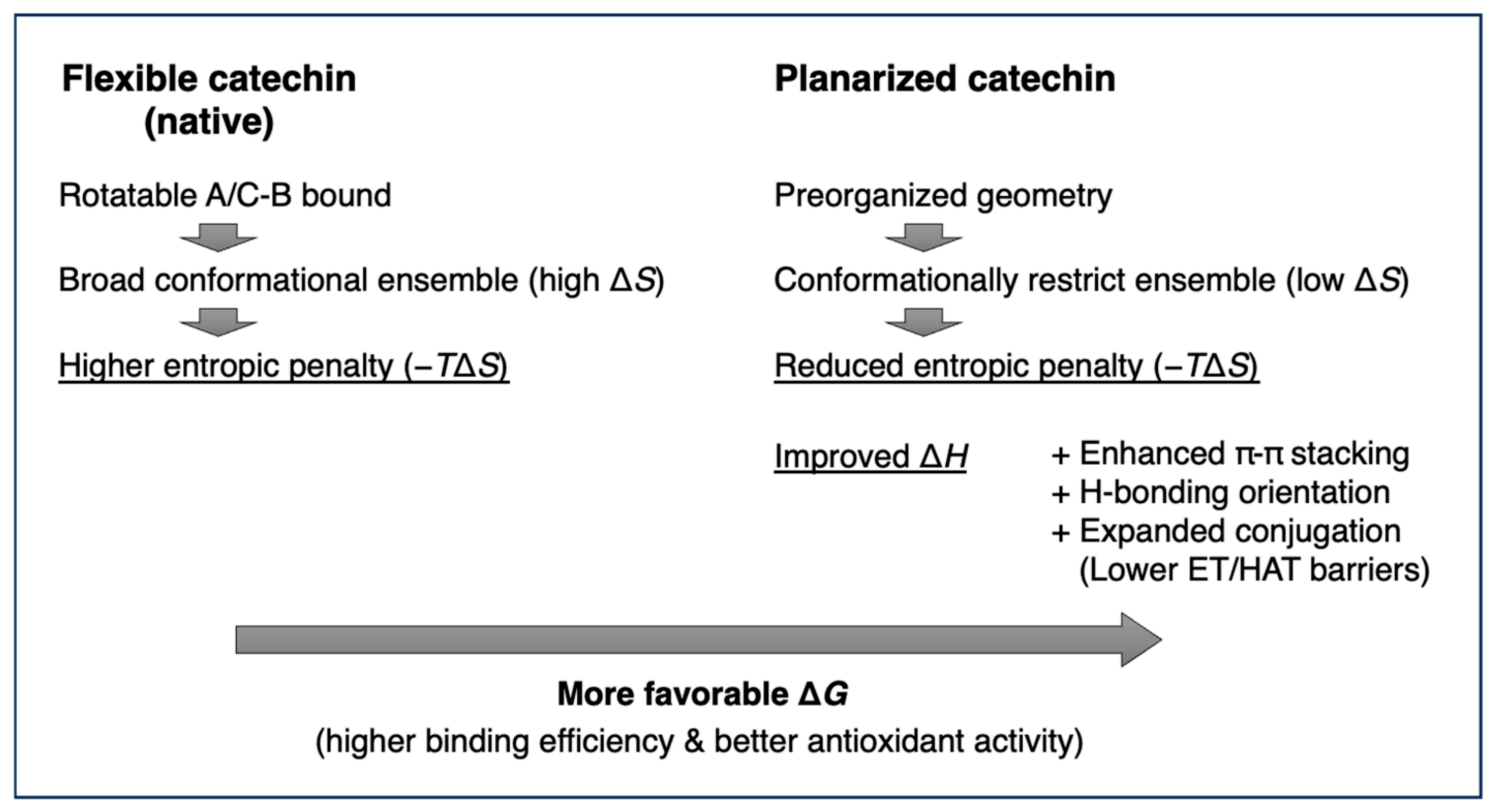
Thermodynamic rationale for planar conformational locking of catechins. Planarization of catechins preorganizes the A/C–B framework, reducing the conformational entropy penalty (−*T*Δ*S*) and enhancing enthalpic interactions (Δ*H*) through expanded π-conjugation, improved π–π stacking, and optimized OH orientation. These enthalpic gains and entropic savings yield a more favorable Δ*G*, underpinning their enhanced antioxidant performance and multiple bioactivities.

Native catechins are flexible flavan-3-ols, and the A/C–B interring bond is a rotatable single bond that yields a broad conformational ensemble in solution. Productive binding typically demands selection and fixation of a minor subset of conformers within the active site, a process that incurs a large conformational entropy penalty (−*T*Δ*S*) and limits how negative Δ*G* can become. By contrast, PCat analogs created through chemical rigidification suppress this torsional freedom ex ante; the preorganized, nearly coplanar A/C–B array reduces the conformational cost paid upon binding, thereby mitigating the entropic penalty and improving the Δ*G* budget even before accounting for enthalpic refinements. In parallel, the enforced planarity expands π-conjugation, aligns aromatic surfaces for stronger π–π stacking and optimized hydrophobic complementarity, and crucially orients B-ring phenolic OH groups so that they more readily satisfy well-ordered hydrogen-bond networks in the protein microenvironment. The net result is a more negative Δ*H* arising from enhanced directionality and cooperativity of noncovalent contacts, superimposed on a smaller −*T*Δ*S* term due to preorganization (Figure 3).

These thermodynamic advantages extend beyond target binding to the elementary steps of redox chemistry that underpin the antioxidant function. The incorporation of the B-ring OH groups into an extended coplanar resonance manifold facilitates spin and charge delocalization in the oxidized states (phenoxyl radicals and radical cations), which stabilizes the relevant transition states and intermediates and lowers the enthalpic barriers for both the HAT and SET–PT pathways. Simultaneously, the preorganized geometry reduces the configurational reorganization required to reach the reactive pose, thereby reducing the entropic cost of the ET/HAT event. In short, planarization delivers a dual benefit, enthalpic gains from strengthened, more directional interactions and entropic gains from reduced conformational search costs, shifting the overall free energy landscape toward more favorable (more negative) Δ*G* for both protein binding and radical-quenching reactions [65,66].

Consistent with this framework, planar catechins exhibit enhanced α-glucosidase inhibition, stronger antiproliferative effects on tumor cells, and markedly improved radical-scavenging performance relative to their flexible counterparts. Part of this improvement can be traced to alkyl substituents installed via bridging chemistry, which fine-tune the microenvironmental complementarity. Their steric and lipophilic profiles promote optimal OH orientation for hydrogen bonding within binding pockets and bias partitioning toward lipid phases where peroxyl radicals are prevalent without reintroducing conformational penalties. Together, these observations underscore the thermodynamic logic of planarization: by preorganizing the catechin scaffold and expanding conjugation, one simultaneously reduces the price of order (−*T*Δ*S*) and increases the value of contact (Δ*H*), achieving a level of binding efficiency and redox effectiveness that is difficult to realize with the native, conformationally labile catechin framework.

## 5. Antiviral Activity of Planarized Catechins

**Figure 4:**
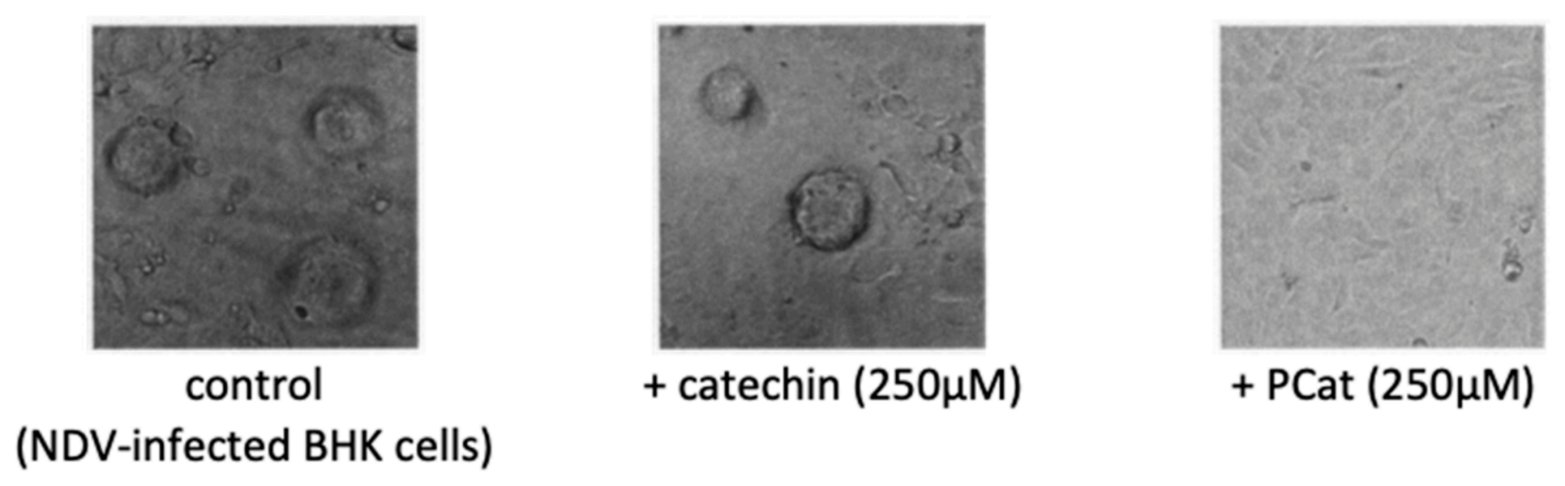
Inhibitory effects of planarized catechin on syncytium formation in NDV-infected BHK cells.

Beyond their canonical redox chemistry, many flavonoid antioxidants, including catechins, exhibit diverse bioactivities, such as antibacterial and antiviral effects [67,68] and modulation of lipid metabolism [69]. However, the potencies of these activities for native catechins are generally modest; by contrast, PCat analogs show clearly superior efficacy, spanning pronounced antiproliferative effects on cancer cells, potent α-glucosidase inhibition [62], robust antiviral activity [70], and strong suppression of radiation-induced apoptosis [63]. With respect to carbohydrase inhibition, PCat displayed potencies comparable to acarbose, a clinically used α-glucosidase inhibitor for type 2 diabetes, and this activity increased monotonically with extension of the alkyl side chain, consistent with a beneficial lipophilicity window for target engagement. Antiviral efficacy was quantified in two BHK-cell assays: (i) cell–cell fusion induced by Newcastle disease virus (NDV) (Figure 4) and (ii) release of infectious virions from vesicular stomatitis virus (VSV)-infected cells. Native (+)-catechin was inactive up to 500 μM, whereas PCat (R = CH_3_) markedly suppressed both NDV-mediated fusion and VSV egress at 250 μM. This activity was further strengthened with increasing alkyl chain length, suggesting that enhanced cellular uptake/partitioning contributed to the observed potency. The parallel structure–activity trends observed for α-glucosidase inhibition imply that the antiviral effects are likely mediated by α-glucosidase inhibition.

Although the precise antiviral mechanism remains to be fully elucidated, the glycoprotein maturation hypothesis is consistent with these data [70]. Specifically, inhibition of endoplasmic-reticulum (ER) α-glucosidase I/II would impede N-glycan trimming on the VSV G glycoprotein, thereby disrupting the calnexin/calreticulin–assisted folding cycle. The resulting ER retention and degradation of misfolded G glycoproteins, together with reduced cell-surface expression and budding, are expected to diminish the formation of infectious particles. This ER-centric mechanism is also in accordance with the observed dependence on lipophilicity, which favors ER access and residency. These antiviral and enzyme-inhibitory phenotypes did not correlate directly with the intrinsic radical-scavenging capacity, indicating that functional derivatization of the planar catechin scaffold can selectively unmask non-redox activities. Taken together, the PCat framework, which is amenable to modular side-chain engineering to tune cell penetration and subcellular localization, provides a medicinally tractable platform for the development of antiviral agents and metabolic modulators that leverage targeted glycoprotein processing interference rather than bulk antioxidant effects.

## 6. Antiproliferative Actions of Planarized Catechins in Cancer Models

Native catechins are widely reported to induce apoptosis in diverse tumor cell types via caspase activation, modulation of the Bcl-2 family, and suppression of NF-κB signaling, while attenuating proliferation, migration, and invasion [30,50,71,72]. These activities support their roles in the primary prevention of dietary/supplemental exposure and suggest their utility as adjuvants at the pharmacological level to enhance chemosensitivity, overcome resistance, and dampen pro-inflammatory signaling. Building on this foundation, a planar catechin (PCat; R = CH_3_) was evaluated in a paired system consisting of a normal rat gastric epithelial line (RGM1) and its carcinogen-transformed counterpart (RGK1). Comprehensive phenotyping encompassed cytotoxicity, mitochondrial membrane potential (Δψm), and two-dimensional migration (scratch assay).

PCat exhibited substantially greater cytotoxicity than (+)-catechin and demonstrated preferential activity toward RGK1 over RGM1, indicating tumor selectivity [73]. At defined concentrations, PCat elicited a decrease in Δψm in cancer cells, consistent with mitochondrial dysfunction–mediated apoptosis. Migration assays further showed that PCat suppressed motility in both cell types, with a more pronounced inhibition of RGK1, indicating interference with redox-dependent cytoskeletal remodeling and signaling pathways that drive tumor cell motility and invasion [74]. Molecular markers aligned with these phenotypes: PCat treatment increased cleaved caspase-3, reduced intracellular ROS accumulation, suppressed NF-κB expression, and induced γH2AX in a dose-dependent fashion [75]. Taken together, these observations support a dual, context-dependent mechanism in which PCat either quenches ROS and down-modulates the NF-κB axis or triggers apoptosis via DNA damage signaling, with the dominant pathway likely dictated by cellular redox state and repair capacity.

From a developmental perspective, the high reactivity of phenolic OH groups raises concerns regarding oxidative inactivation and off-target reactions in biological matrices. To address this, acetylated planar catechin (Ac-PCat; masking phenols) was prepared to enhance chemical stability [76]. Ac-PCat displayed greater cytotoxic potency in cancer cells than the parent PCat yet showed reduced radical-scavenging activity in cell-free assays, as expected for a protected phenol. Exposure to intracellular esterases restored phenolic function, and ESR measurements confirmed the recovery of superoxide-scavenging activity following deacetylation. These data are consistent with a prodrug-like mechanism, wherein acetylation shields the redox pharmacophore during distribution, and enzymatic unmasking within cells regenerates the active phenolic network that supports both antioxidant and pro-apoptotic outcomes.

**Figure 5:**
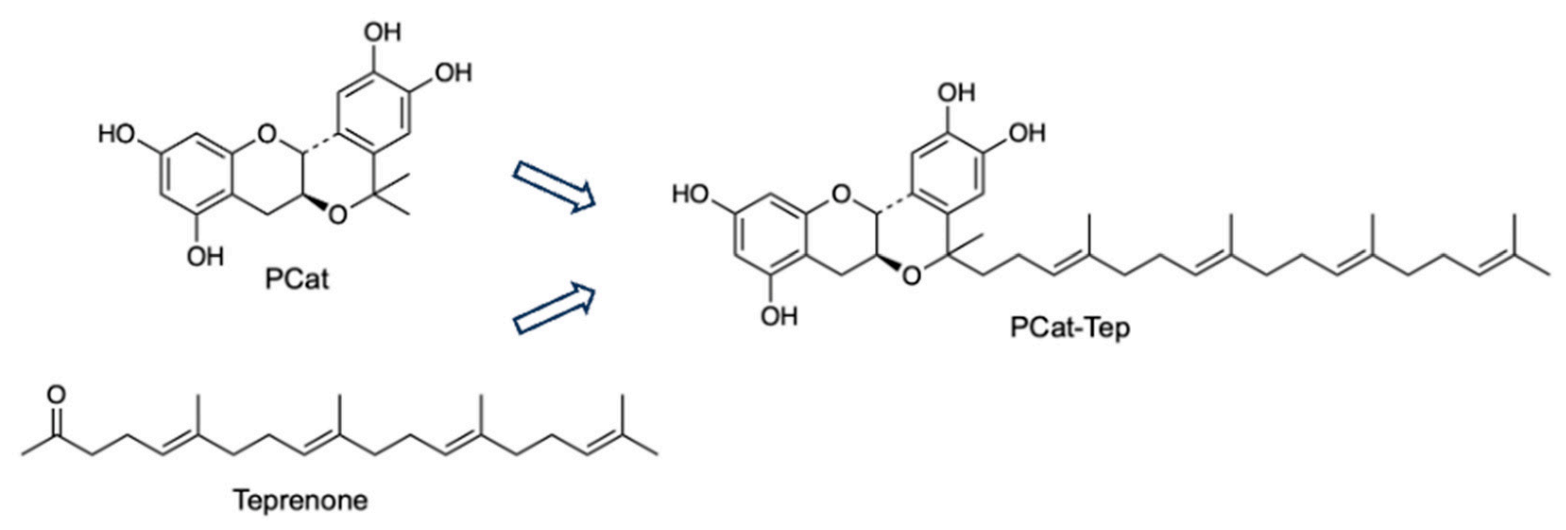
PCat–Tep hybrid via covalent fusion of planar catechin with teprenone.

In aggregate, conformationally locked, planar catechins integrate potent antioxidant capacity with tumor-biased cytotoxicity and migration suppression and, through phenol masking, offer a controllable activation switch that improves stability without forfeiting intracellular efficacy. This constellation of properties positions PCat scaffolds as promising chemical platforms for cancer prevention and therapy, enabling modality-agnostic combinations (e.g., with DNA-damaging agents or NF-κB inhibitors) and motivating continued structural elaboration to tune lipophilicity, phenol p*K*a, and deprotection kinetics to balance selectivity, depth of response, and safety. Extending this design logic, a PCat–teprenone (PCat–Tep) conjugate was further developed to test whether membrane-targeting elements could amplify potency via complementary delivery and redox mechanisms (Figure 5) [77]. Exploiting the ketone functionality of teprenone (Tep), PCat–Tep was assembled by OPS cyclization between (+)-catechin and teprenone, thereby locking the linkage within a conformationally constrained framework. Tep, a gastroprotective drug known to induce heat shock proteins and thioredoxin, has been associated with redox regulation and cytoprotection in gastric mucosal cells [78,79]. Based on these considerations, covalent fusion of PCat with this terpene-like, lipid-affine unit was expected to increase intracellular delivery and amplify apoptotic output beyond either component alone. Indeed, PCat–Tep exerted marked cytotoxicity in RGK1 cells at concentrations where PCat or a PCat + Tep mixture was minimally active, underscoring the importance of covalent linkage. Exposure to 25 μM PCat–Tep robustly induced Annexin V expression and caspase-3 activation, indicative of apoptosis. These findings suggest that the Tep moiety facilitates membrane association and local accumulation of PCat, yielding synergistic amplification of its intrinsic cytotoxicity.

## 7. Structural Modifications to Amplify the Antioxidant Performance of Planarized Catechins

### 7.1. PCat–Lys Conjugate

**Figure 6:**
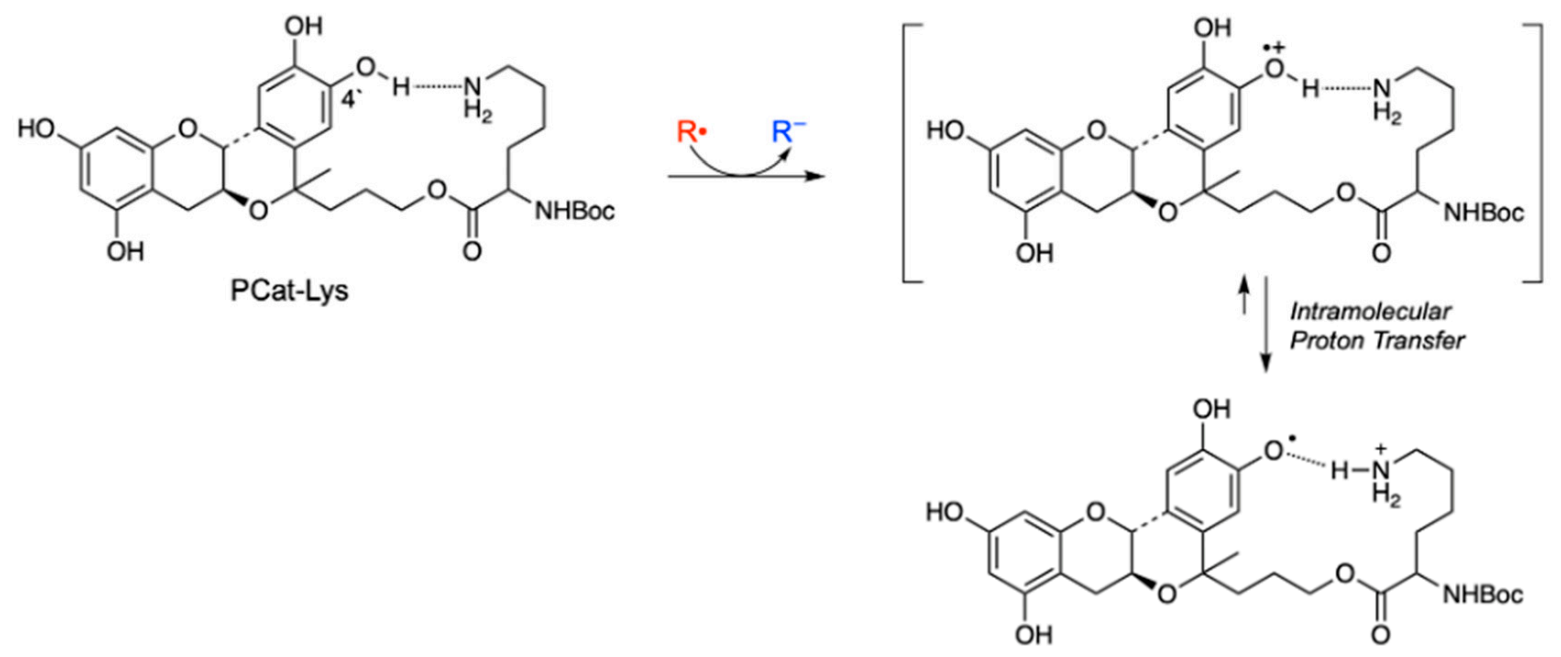
Radical scavenging mechanism of PCat–Lys conjugate: Stabilization of the radical cation by the lysine side-chain amino group.

Phenolic radical trapping by catechins is markedly accelerated under basic conditions [59,80], a kinetic effect commonly attributed to the stabilization of transient radical cations formed along the scavenging coordinate. Based on this mechanistic rationale, embedding a basic site intramolecularly was hypothesized to reproduce this stabilization at neutral pH, thereby enhancing radical-scavenging activity without relying on the basicity of the bulk solution. Guided by this concept, a planar catechin–lysine conjugate (PCat–Lys) was designed in which a linker of optimized length positions the ε-amino group proximal to the 4′-OH of the B ring (Figure 6) [81]. Computation-guided spacing enabled intramolecular hydrogen bonding between the 4′-OH proton and the lysyl nitrogen, preorganizing the conjugate to stabilize oxidized intermediates.

Kinetic measurements revealed a ~420-fold increase in the radical-trapping rate constant (*k*) relative to that of catechin. Quantum-chemical optimization of the PCat–Lys radical cation corroborated the mechanistic model, showing proton transfer from the 4′-OH to the proximal amine, which markedly stabilizes the oxidized state and lowers the barrier for H-atom or electron transfer. Extending this concept, arginine- and histidine-tethered PCat analogs also exhibited strong rate enhancements, and their *k* values correlated with the basicity (p*K*a) of the appended amino acid side chain. Together, these data established that intramolecular basic assistance is a general strategy for amplifying catechin-based radical scavenging under physiological conditions.

### 7.2. PCat–TrOH Hybrid

**Figure 7:**
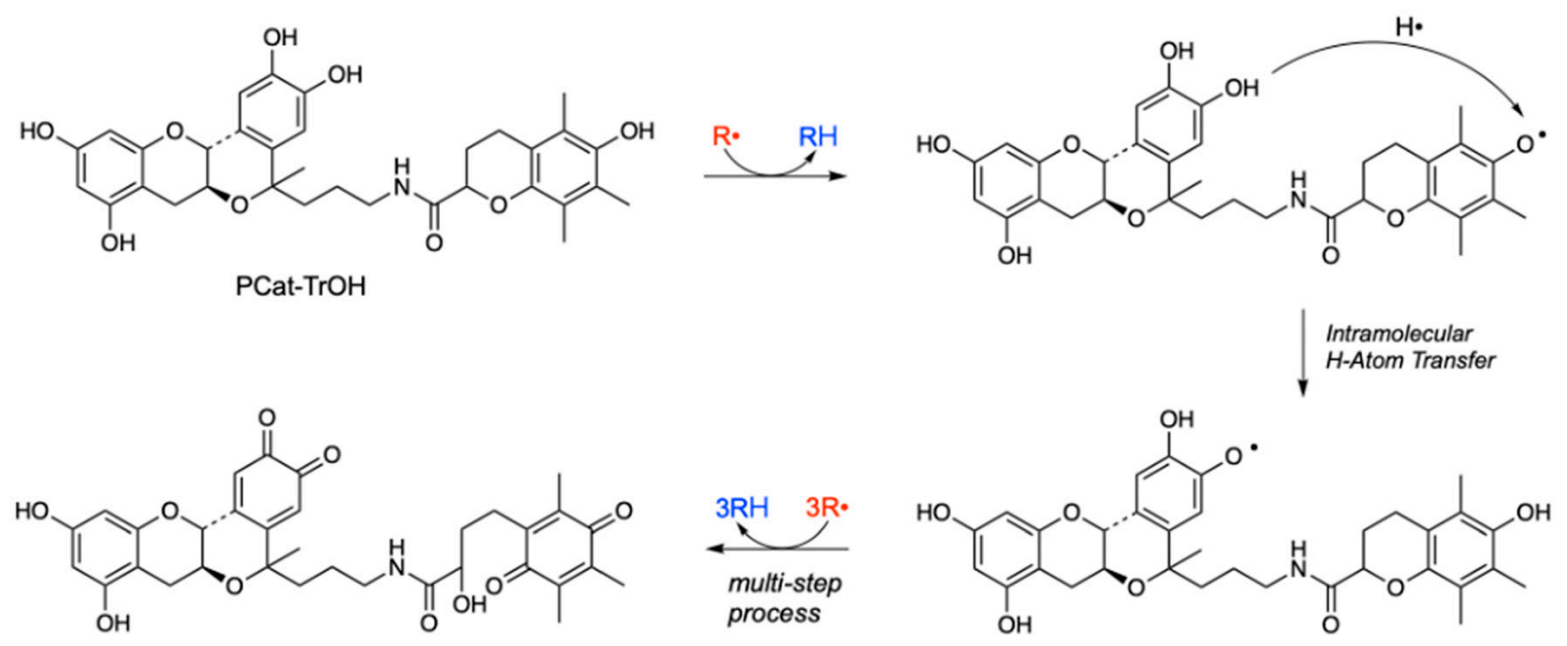
Radical scavenging mechanism of PCat–TrOH: Intramolecular H-atom transfer from PCat to TrO^•^.

Lipid peroxidation propagates via radical chain reactions in polyunsaturated membranes and is normally curtailed by lipophilic antioxidants such as α-tocopherol; oxidized tocopherol is then regenerated by vitamin C, which itself is recycled by glutathione and NAD(P)H, constituting an antioxidant network [82,83]. To emulate this concerted regeneration within a single molecular framework, a hybrid antioxidant was designed in which planar catechin was covalently integrated with trolox (TrOH), the chromanol moiety that embodies the radical-scavenging core of α-tocopherol, yielding the dual-acting PCat–TrOH construct (Figure 7) [84]. Although TrOH is ~6.5-fold faster than PCat in radical trapping, PCat–TrOH retains the kinetic potency of TrOH while doubling the total radical equivalent capacity relative to either component alone. The data are consistent with a self-regenerating intramolecular cycle, in which PCat and TrO^•^ exchange H/*e^−^* (PCat ⇄ TrO^•^) to sustain activity; the hybrid thus improves both the rate (“activity”) and the equivalents (“capacity”) of scavenging on a unified scaffold.

Membrane partitioning by the PCat core, together with the regenerability of the TrOH site, suggested that PCat–TrOH could reinforce lipid chain breaking in concert with ascorbate cross-talk, analogous to α-tocopherol recycling. Ongoing studies are quantifying the suppression of lipid peroxidation in cell and bilayer models, the ascorbate-driven regeneration efficiency, and the ESR-resolved behavior of radical intermediates. As a design prototype, PCat–TrOH integrates planarity-enhanced redox performance with a built-in regeneration relay, providing a blueprint for next-generation lipophilic antioxidants that co-optimize kinetics and capacity on a single-molecule platform.

### 7.3. PCat–Diethylenetriaminepentaacetic Acid (DTPA): Fe^3+^-Triggered Antioxidant Activity

**Figure 8:**
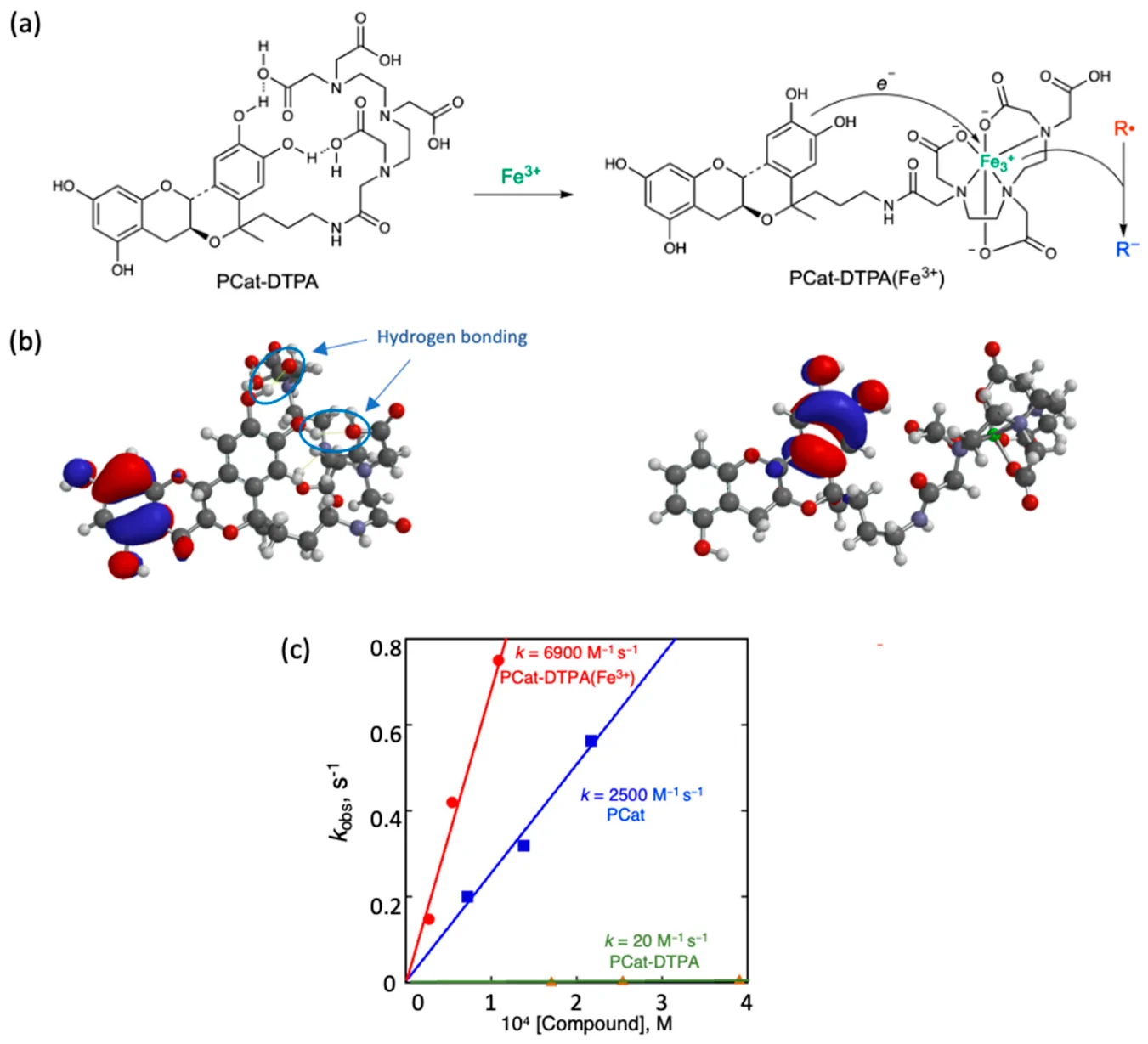
Fe^3+^-triggered radical scavenging in a planar catechin–DTPA conjugate. (**a**) Fe^3+^ coordination switches PCat–DTPA “ON,” enabling Fe^3+^-mediated one-electron transfer to R• (ROS). (**b**) DFT-optimized lowest-energy structures of PCat–DTPA and its Fe^3+^ complex. The apo form features an intramolecular H-bond, and Fe^3+^ binding relocates the HOMO to the catechol unit. (**c**) Pseudo-first-order rate constant (*k*_obs_) as a function of the concentration of PCat (blue), PCat–DTPA (green), and PCat–DTPA(Fe^3+^) (red) for DPPH^•^ radical scavenging.

Sites of oxidative stress pathology frequently accumulate redox-active metals (e.g., iron) that catalyze ROS production via Fenton/Haber–Weiss chemistry [85,86]. Conventional polyphenols scavenge ROS but do not adequately control metal-mediated catalysis in lesions [87]. To couple metal sequestration with on-demand radical trapping, PCat–DTPA, a planar catechin conjugated to the chelator DTPA, was synthesized (Figure 8a) [88]. In the resting state, intramolecular H-bonding (IHB) between catechol and DTPA shifts the HOMO toward the A ring, effectively locking electron donation and reducing the radical-scavenging activity to approximately 1/10 that of PCat. Upon Fe^3+^ coordination, this IHB is disrupted, electron density is redirected back to the catechol, and the metal ion serves as an electron-relay hub (Figure 8b). Because of Fe^3+^ coordination, the resulting PCat–DTPA·Fe^3+^ complex exhibits a ~3.7-fold enhancement in radical-scavenging activity relative to PCat (Figure 8c).

Mechanistically, the system parallels the intramolecular network inferred for PCat–TrOH but is triggered by Fe^3+^ binding: inner-sphere electron transfer from the catechol generates PCat^•^ and Fe^2+^, after which the coordinated Fe^2+^ performs outer-sphere electron transfer to reduce and deactivate surrounding radical species. Critically, iron remains securely chelated by DTPA, minimizing leakage of free Fe^2+^ and the attendant risk of ^•^OH generation. Thus, PCat–DTPA simultaneously quenched the source of ROS (metal catalysis) and removed the formed radicals, delivering a lesion-selective, high-efficiency antioxidant action. Because the conjugate is quiescent in normal tissues, the risk of pro-oxidant behavior is inherently low; activation is conditional on Fe^3+^, conferring pathology-selectivity.

From a medicinal chemistry standpoint, ADME optimization, such as ester-prodrug strategies to enhance lipophilicity and uptake, represents a logical next step. Overall, PCat–DTPA emerged as a chemically tractable lead for metal-associated oxidative stress disorders with triggered activity, metal removal, and planarity-enabled redox efficiency within a single modular scaffold.

## 8. Conformational Fixation of Natural Products Through Planarized Catechin Installation

### 8.1. Procyanidins

**Figure 9:**
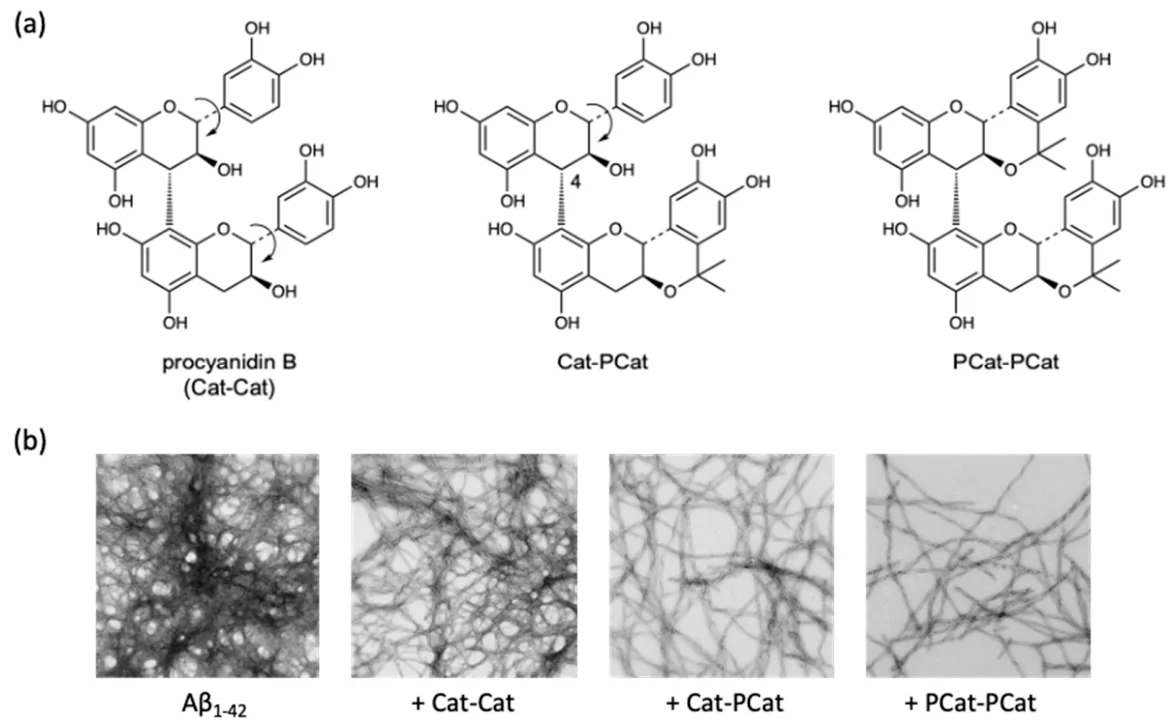
(**a**) Procyanidin B and its conformationally planarized derivatives (Cat–PCat, PCat–PCat). (**b**) TEM images of Aβ_1–42_ aggregation in the presence of planarized procyanidin analogs.

Procyanidins are oligomeric/polymeric condensates of catechins that are abundant in apples, grapes, and cacao and are credited with broad bioactivities, including potent antioxidant capacity, anti-atherogenic effects, and improvements in lipid and glucose metabolism [89,90,91]. In the context of AD, multiple studies report that procyanidins impede the pathogenic self-assembly of Aβ by suppressing the formation of neurotoxic oligomers and subsequent fibrillar aggregates [92,93,94]. Nevertheless, highly polymerized species display poor blood–brain barrier (BBB) permeability owing to their size and polarity, constraining their translational potential in AD. To address this limitation, procyanidin B3 (Cat–Cat), a low-molecular-weight dimer reported to traverse the BBB, was selected as the scaffold for conformational fixation to co-enhance antioxidant potency and Aβ aggregation inhibition [95,96]. Accordingly, two conformationally locked dimers were synthesized: (i) Cat–PCat, in which one catechin unit was planarized by first introducing a 4-position leaving group on catechin and then condensing PCat onto this handle, and (ii) PCat–PCat, in which both units were planarized by reacting phenol-protected B3 with acetone under Lewis acid catalysis (Figure 9a). In the radical-trapping assays, Cat–Cat was ~3.8-fold more potent than monomeric catechin; Cat–PCat improved 1.9-fold over Cat–Cat (~7-fold vs. catechin), and PCat–PCat added another 1.5-fold over Cat–PCat (~11-fold vs. catechin). The activity ranking correlated with decreasing ionization potential (IP), consistent with π-conjugation expansion upon planarization and the attendant increase in electron-donor strength.

Against Aβ, all dimers outperformed monomeric catechin in aggregation suppression, with planarization amplifying efficacy and PCat–PCat showing the strongest inhibition. (Figure 9b) [97]. Mechanistically, the dimers plausibly interfere with early β-sheet assembly through multivalent contacts, including (i) competitive π–π interactions with the hydrophobic core (proximal to aromatic residues such as Phe19/Phe20), (ii) hydrogen-bonding mediated by catechol groups, and (iii) geometric complementarity provided by the enlarged coplanar π-surfaces. Concordantly, Aβ-induced neurotoxicity was significantly attenuated, with PCat–PCat affording the most pronounced protection. The dual action, antioxidant quenching together with aggregation blockade, is likely synergistic, as suppression of Aβ-derived ROS helps blunt downstream toxic signaling (e.g., Ca^2+^ dysregulation and oxidative-stress responses). While the potent radical-scavenging action of theaflavins—another class of catechin-derived oxidative dimers—derives from a complex network of intramolecular interactions stemming from their specific conformation [98], it is conceivable that similar intramolecular interactions also contribute to the strong radical-scavenging activity observed in procyanidins and their conformationally fixed monomeric/dimeric catechin counterparts. Overall, planar fixation of procyanidin B3 achieves simultaneous enhancement of redox kinetics/capacity and Aβ antagonism/neuroprotection by strengthening electron donation and optimizing multisite molecular recognition. Retaining a dimeric, BBB-permissive size class, PCat–PCat emerges as a promising scaffold to target both Aβ-driven oxidative stress and amyloid pathology in the AD brain.

### 8.2. Silybin

**Figure 10:**
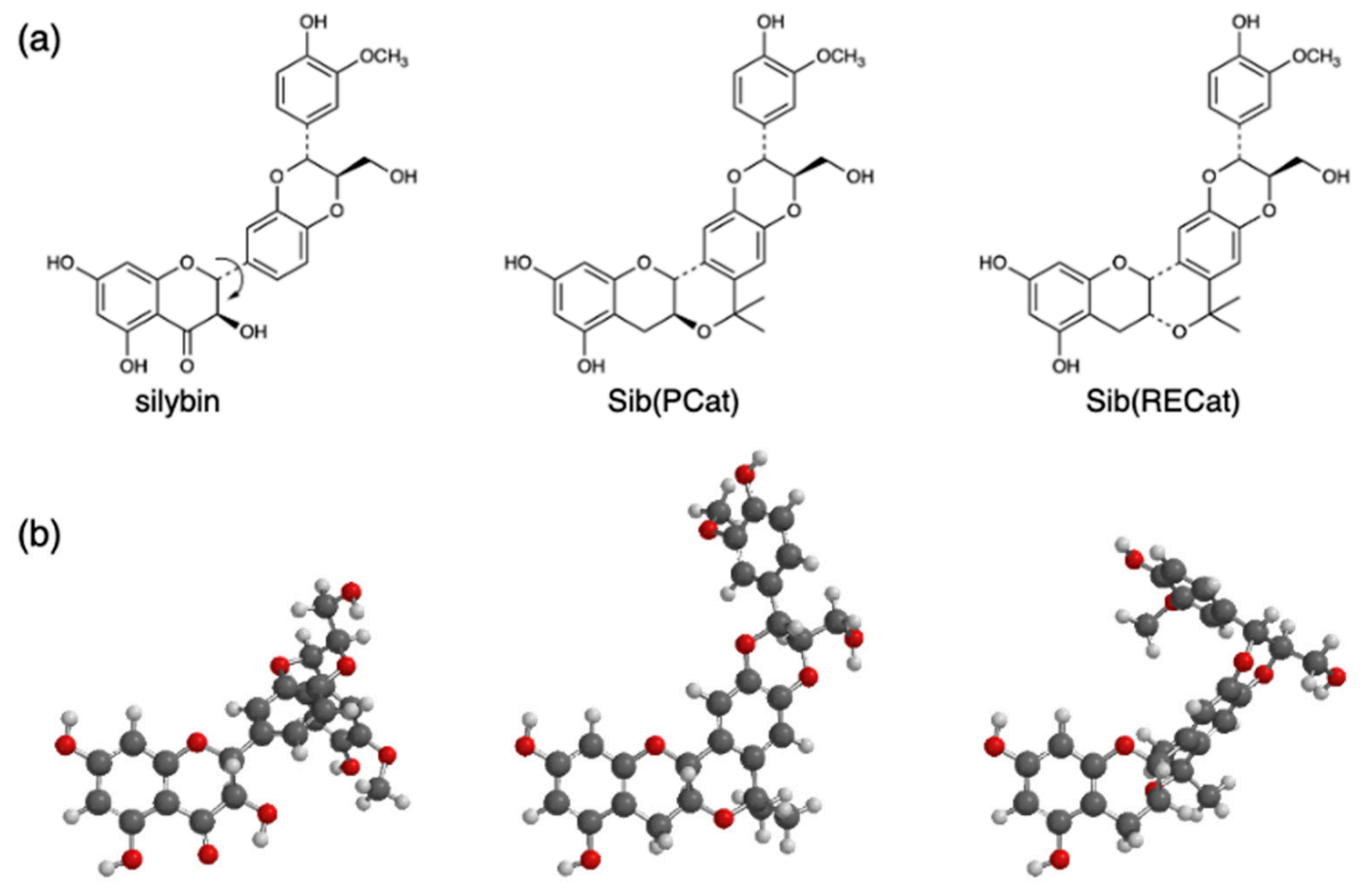
Silybin and conformationally rigidified analogs. (**a**) Structures of silybin and its derivatives, Sib(PCat) and Sib(RECat). Sib(PCat) contains an installed planar catechin (PCat) module, whereas Sib(RECat) incorporates a conformationally rigid epicatechin (ECat) module into the silybin scaffold. (**b**) DFT-optimized lowest-energy geometries of the above compounds.

Silybin (silibinin), a flavanonol found in milk thistle seeds, exhibits hepatoprotective, anticancer, and antidiabetic activities and is widely used as a dietary supplement [99,100,101,102]. More recently, silybin has been shown to inhibit Aβ aggregation and mitigate neurotoxicity in AD models [103,104,105]. Structurally, silybin contains several rotatable C–C bonds within a flexible flavanonol framework; therefore, productive binding to biomolecular targets entails an induced fit with an attendant entropic penalty. To minimize this conformational liability, Sib(PCat), a silybin analog in which the conformationally mobile segment was replaced with a PCat module, was designed to preorganize the molecule in its putative active conformation (Figure 10) [106]. For comparison, Sib(RECat), in which the PCat module was replaced with a conformationally rigidified epicatechin (RECat) insert generated via OPS cyclization, was also prepared to intentionally distort the parent geometry. Functional evaluation showed that Sib(PCat) exhibits markedly stronger Aβ anti-aggregation activity than native silybin. In addition, α-glucosidase inhibition and growth suppression were significantly enhanced in the selected cancer cell lines. In comparison, Sib(RECat) displayed substantially reduced activity across these assays, indicating that targeted planar fixation, rather than arbitrary conformational alteration, is optimal for amplifying the pleiotropic bioactivities of silybin. Mechanistically, the PCat insertion likely lowers oxidation potential, expands π-surface area for favorable π–π/hydrophobic contacts, and reduces conformational entropy loss on binding, thereby improving both enthalpic complementarity and entropic efficiency.

Altogether, these case studies, procyanidin dimers, and silybin derivatives demonstrate that replacing conformationally mobile segments with PCat modules to preorganize the scaffold is a general and powerful strategy that simultaneously enhances antioxidation, anti-Aβ aggregation, and enzyme-inhibition/cell-modulatory activities. Thus, conformational fixation represents a versatile lead optimization strategy, providing chemically tractable entry points for medicinal chemistry and a coherent blueprint for advancing natural-product-derived therapeutics.

## 9. Conclusions

Plants live under persistent oxidative pressure and have evolved endogenous antioxidants to mitigate the damage caused by ROS and free radicals. Appropriately leveraging these natural defenses through dietary intake or pharmacological translation is a rational approach for preventing lifestyle-related disorders and age-associated functional decline. Contemporary evidence establishes that natural antioxidants act far beyond simple radical quenchers; they engage antioxidant networks and modulate inflammatory and proteostatic pathways (anti-amyloid actions), thereby contributing to disease prevention and therapy.

From the perspective of drug discovery, natural products combine structural diversity, biocompatibility, and critically favorable safety. Phenolic frameworks such as catechins, which are already used as supplements in humans, are attractive starting points. Optimization of ligand binding requires control of the free energy of interaction with target proteins, while entropic tuning is notoriously difficult; modern design emphasizes enthalpy-driven recognition. PCat addresses both terms simultaneously: preorganization of conformation reduces the entropic penalty upon binding, while planarity and tuned donor strength deepen enthalpic contacts through extended π-surfaces, directed H-bonding, and favorable electrostatics.

The case studies surveyed here, PCat–DTPA (a lesion-activated antioxidant/metal-control hybrid triggered by Fe^3+^), PCat–TrOH (a self-regenerating intramolecular antioxidant network), procyanidin B3 derivatives (Cat–PCat and PCat–PCat), and the silybin analog Sib(PCat), collectively validate the design triad of planarization × multivalent recognition × electronic tuning. Across orthogonal assays (anti-amyloid aggregation, enzyme inhibition, and cellular function), this principle delivered robust and context-appropriate activity gains.

As the pathogenesis of oxidative stress varies across diseases, initiating chemistry (metal catalysis, lipid radical chains, and mitochondrial leakage) and operative species vary, therapeutics must be matched to the mechanism. Phenolic antioxidants differ in their site of action, mode, and ROS selectivity as a function of their structure. Effective prevention and treatment, therefore, demand mechanism-informed molecular tailoring: dissect the operative ROS circuitry and then introduce the minimal, essential modifications that deliver “the right function, at the right site, at the right intensity.” In this regard, PCat provides a modular, high-functionality scaffold. Chelators, radical-trapping units, and oriented hydrophobic or charged groups can be combined to allocate functions precisely. Practically, beginning with a widely available, supplement-familiar core streamlines synthesis and de-risks development, facilitating favorable safety profiles.

Cumulatively, this review shows that conformational fixation of catechins coupled with rational substituent engineering can sharpen the diffusion of native activities into disease-relevant capabilities, enhancing binding affinity, enabling conditional activation, and improving target access. Advancing a PCat-centered, modular strategy aligned with the disease mechanism should enable natural-antioxidant-based discovery to move from promise to practice and deliver genuine breakthroughs in new drug development.

## Data Availability

No new data were created or analyzed in this study. Data sharing is not applicable to this article.

## Abbreviations

The following abbreviations are used in this manuscript:

Aβ	amyloid-β
AD	Alzheimer’s disease
BBB	blood–brain barrier
BDE	bond dissociation enthalpy
ER	endoplasmic reticulum
ET	electron transfer
HAT	hydrogen atom transfer
IHB	intramolecular H-bonding
IP	ionization potential
NDV	Newcastle disease virus
OPS	oxa–Pictet–Spengler
PCat	planar catechin
ROS	reactive oxygen species
SPLET	sequential proton loss electron transfer
SET-PT	single-electron transfer followed by proton transfer
Tep	teprenone
TrOH	trolox
VSV	vesicular stomatitis virus
